# How to take a complete eye history

**Published:** 2019-12-17

**Authors:** Moureen Takusewanya

**Affiliations:** 1 Ophthalmologist: Mbarara Hospital, Uganda.

**Taking a good history not only helps you to make a diagnosis, it can also help you to understand the impact of the condition on the patient and identify any obstacles to treatment.**

**Figure:**
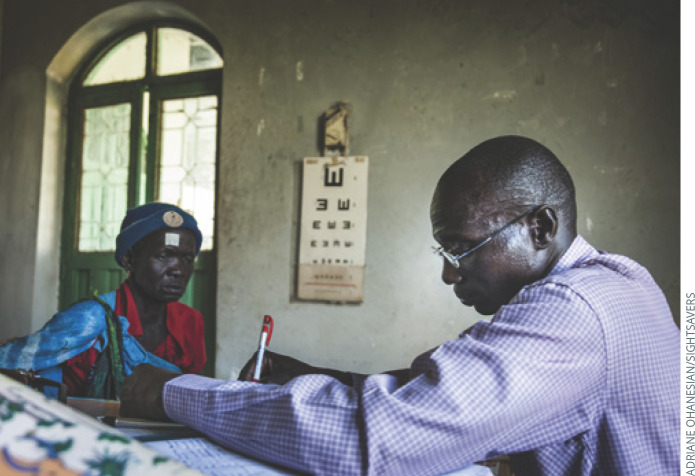
Make careful notes when you take a history. SOUTH SUDAN

It is impossible to over-emphasise the importance of taking a careful history when assessing an eye patient. Taking a good history can help to focus your examination and indicate what investigations are needed. It can also help you to understand the impact of the condition on the patient and pinpoint any difficulties they may have adhering to treatment.

This is also your opportunity to focus on the patient as a person and to form a relationship of trust, respect and mutual understanding.

## How to structure history taking

To ensure you don't miss anything important, structure your history taking carefully. Ask about:

- Personal and demographic data
- Reason for visit or presenting complaint
- History of presenting complaint
- Past eye history
- General medical history
- Family eye history
- Medication history
- Allergy history
- Social history

**Tip:** You can use this bulleted list as a checklist Copy and keep it where you can see it during history taking to help you to stay on track and ensure that you will not miss anything important.

Each of these is discussed in more detail below.

**Top tips for taking a good history**

- Introduce yourself to the patient – this creates a friendly environment.
- Respect the patient's privacy and confidentiality while taking the history
- Ask questions that are direct, simple and clear. Avoid using medical terms and explain things in ordinary language as much as possible.
- Be a good listener. Avoid interrupting or rushing the patient. Show them that you are listening and paying attention: make eye contact as appropriate and ask if you are not sure about something they said. It is often useful to use open questions (e.g., how are you?) and closed questions (e.g., yes/no answers) to help focus the discussion.
- Try to see things from the patient's point of view and make an effort to understand them and their circumstances, especially when these are very different from your own.
- Be aware that patients who are older, or who have disabilities (including hearing impairment, speech difficulties or a learning disability) may need a bit more time or may struggle to express themselves. This may cause them some anxiety, so remain patient and reassure them that you are there to listen.

### Personal and demographic data

Ask the patient's personal details:

- Name, for identification, filing and patient follow-up
- Address and mobile phone number, for follow-up and to identify patients from areas with endemic diseases
- Age and gender, for noting down and ruling out any diseases associated with different age groups and/or sex
- Language
- Disability
- Patient's occupation, daily tasks and hobbies.

Recording the age, gender, language and disability status of patients allows you to monitor who is, and is not, coming to your eye clinic or hospital. Compare these figures with the population to identify groups that are under-represented, e.g., girls with other disabilities, and plan ways to reach out to them.

Understanding a patient's occupation, daily tasks (e.g., looking after grandchildren) and hobbies is helpful for finding out a patient's visual needs and understanding any eye manifestations or symptoms as a result of occupational hazards.

### Reason for visit/Presenting complaint

Ask the main reason why the patient has come to seek an eye examination.

Record the main presenting symptoms in the patient's own words and in a chronological order. The four main groups of symptoms are:

1. Red, sore, painful eye or eyes (including injury to the eye)
2. Decreased distance vision in one or both eyes, whether suddenly or gradually
3. A reduced ability to read small print or see near objects after the age of 40 years
4. Any other specific eye symptom, such as double vision, swelling of an eyelid, watering or squint.

### History of presenting complaint

This is an elaboration of the presenting complaint and provides more detail. The patient should be encouraged to explain their complaint in detail and the person taking history should be a patient listener. While taking a history of the presenting complaint, it is important to have potential diagnoses in mind. For each complaint, ask about:

- Onset (sudden or gradual)
- Course (how it has progressed)
- Duration (how long)
- Severity
- Location (involving one or both eyes)
- Any relevant associated symptoms
- Any similar problems in the past
- Previous medical advice and any current medication.

### Past eye history

Ask for detail about any previous eye problems

- **History of similar eye complaints** in the past. This is important in recurrent conditions such as herpes simplex keratitis, allergic conjunctivitis, uveitis and recurrent corneal erosions
- **History of similar complaints in the other eye** is important in bilateral conditions such as uveitis, cataract
- **History of past trauma to the eye** may explain occurrence of conditions such as cataract and retinal detachment
- **History of eye surgery.** It is important to ask about any ocular surgery in the past such as cataract extraction, muscle surgery, glaucoma, or retinal surgery
- **Other symptoms**. Ask whether the patient has any other specific eye symptoms.

### General medical history

Ask about any current and past medical conditions. These include conditions such as diabetes, hypertension, arthiritis, HIV, asthma and eczema.

### Family eye history

It is important to ask the patient whether any other member of the family has a similar condition or another eye disease. This can help to establish familial predisposition of inheritable ocular disorders like glaucoma, retinoblastoma or congenital eye diseases, diabetes and hypertension.

### Medication history

Ask about present and past medications for both ocular and medical conditions. Don't overlook any medications that the patient may have stopped taking some time ago. Some medications are important in the etiology of ocular conditions.

It is also helpful to ask whether the patient has been able to use the medication as prescribed (their compliance). If a medication is ineffective, you want to know whether the patient is actually using the medication as prescribed, for example glaucoma medications.

Using your own discretion, it is helpful to find out whether access to medication prescribed is a problem. This helps to ascertain whether cost or other concerns are a potential reason for non-compliance. There could also be practical issues, such as difficulty instilling eyedrops or forgetting to do so.

Do not forget to ask in a non-judgmental way about traditional/herbal medication use.

**Figure 1:**
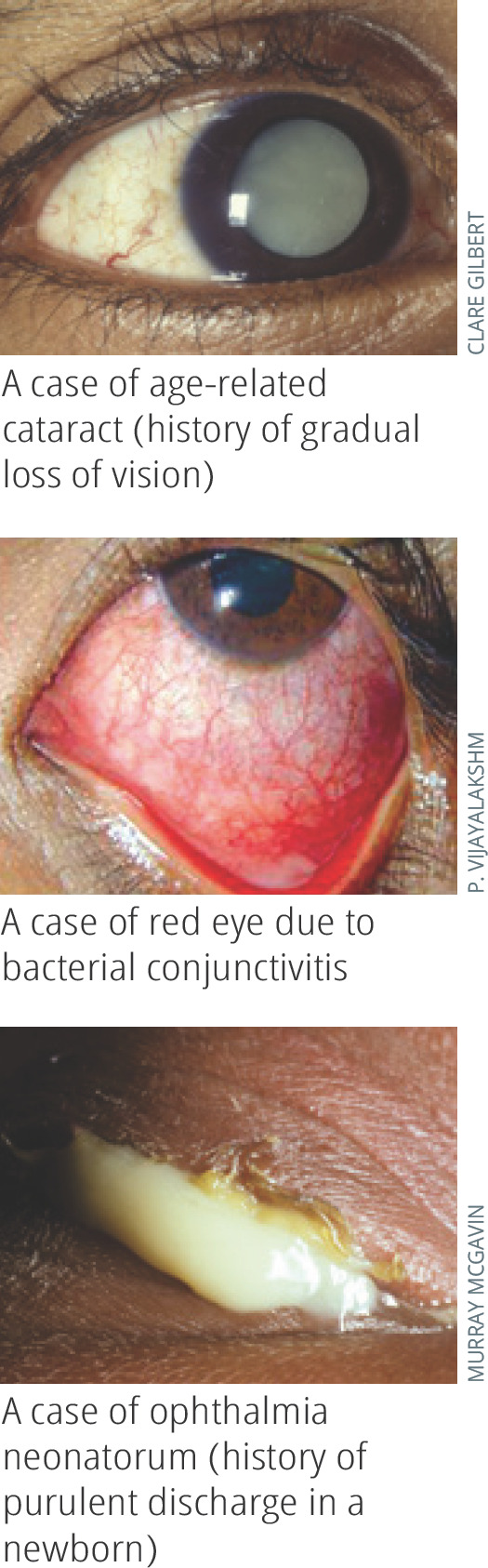
Case scenarios with different presenting complaints

### Allergies

Ask about any allergies to medications or other substances.

### Social history

- Smoking (amount, duration and type)
- Alcohol (amount, duration and type)

### Birth and immunisation history

For children, the birth history (prematurity) and immunisation status can be important.

